# Association of resistance training and moderate-to-vigorous physical activity with clinical outcomes in men with airflow limitation: a nationwide population-based study

**DOI:** 10.1038/s41598-024-57232-6

**Published:** 2024-03-18

**Authors:** Taeyun Kim, Seok Min Hyun, Sun Hye Shin, Yunjoo Im, Yoonju Na, Jong Geol Do, Hye Yun Park, Sunga Kong

**Affiliations:** 1 Division of Pulmonary, Department of Internal Medicine, Kosin University Gospel Hospital, Kosin University College of Medicine, Busan, Republic of Korea; 2 Division of Pulmonary and Critical Care Medicine, Department of Internal Medicine, Samsung Medical Center, Sungkyunkwan University School of Medicine, 81 Irwon-Ro, Gangnam-Gu, Seoul, 06351 Republic of Korea; 3 Department of Physical and Rehabilitation Medicine, Samsung Medical Center, Sungkyunkwan University School of Medicine, Seoul, Republic of Korea; 4 Department of Clinical Research Design and Evaluation, SAIHST, Sungkyunkwan University, 115 Irwon-Ro, Gangnam, Seoul, 06335 Republic of Korea

**Keywords:** COPD, Resistance training, MVPA, Exercise, Cough, Sputum, Sleep, Chronic obstructive pulmonary disease, Quality of life

## Abstract

Aerobic moderate-to-vigorous physical activity (MVPA) is recommended for individuals with chronic diseases. However, the association between resistance training (RT) in addition to moderate to vigorous physical activity (MVPA) and sleep duration, as well as respiratory symptoms, in patients with chronic obstructive pulmonary disease has not been thoroughly investigated. This population-based cross-sectional study used data from the Korea National Health and Nutrition Examination Survey between 2014 and 2019. A total of 61,754 individuals were identified and men with airflow limitation (FEV_1_/FVC < 0.7) who engaged in aerobic MVPA were selected (n = 794). Weighted percentages and odds ratio (OR) of sleep problems (≤ 5 or ≥ 9 h), chronic cough, and chronic sputum were estimated. A multivariate-adjusted complex sample logistic regression model was used to calculate ORs and 95% confidence intervals (CI). Subgroup analyses were conducted using the forced expiratory volume (FEV_1_) % of the predicted value (%pred) ≥ 80 vs. < 80. The percentages of sleep problems, chronic cough, and chronic sputum production were lower in men who underwent aerobic MVPA + RT than in those who underwent aerobic MVPA alone. The multivariable-adjusted OR of sleep problems was 0.44 (95% CI 0.25–0.77) in individuals undergoing aerobic MVPA + RT compared to aerobic MVPA alone. The ORs of chronic cough and sputum were 0.35 (95% CI 0.13–0.94) and 0.51 (95% CI 0.30–0.87), respectively. These associations were only significant in individuals with FEV_1_ < 80% pred. Compared with aerobic MVPA alone, aerobic MVPA + RT was associated with appropriate sleep duration and a decrease in chronic cough and sputum in male with airflow limitation. This was more pronounced in individuals with a FEV_1_ < 80% pred**.**

## Introduction

Chronic obstructive pulmonary disease (COPD) causes chronic respiratory symptoms with persistent and often progressive airway obstruction. It is a major cause of mortality and healthcare-use worldwide^1^. Reducing symptoms, optimizing health status, and promoting daily physical activity (PA) constitute major goals in COPD management^2^. Cough and sputum are two of the most uncomfortable respiratory symptoms in patients with COPD. Higher levels of these symptoms are associated with poor patient-reported outcomes, including physical function, fatigue, and insomnia^3^. Night sleep disturbance is more frequently reported in patients with COPD most affected by cough and sputum^3–5^. Given the close relationship between respiratory symptoms and sleep quality, this vicious circle could plausibly explain the daily limitations of PA in patients with COPD^6,7^.

PA refers to any bodily movement that requires energy expenditure and engages muscles. PA level is generally lower in patients with COPD than in healthy older individuals^8^. Chronic respiratory symptoms, sedentary behavior, muscle weakness, exercise incapacity, and physical deconditioning unitedly create a downward disease spiral in COPD^9–12^. The Global Initiative for COPD (GOLD) 2023 recommends all patients to be physically active. However, PA above a certain threshold, moderate-to-vigorous PA (MVPA), is required to attain improvement^13^. Indeed, data from a large population-based cohort revealed that MVPA is inversely related to symptom burden and mortality in patients with COPD^14,15^.

The World Health Organization in 2020^16^ and the PA guidelines for Americans in 2018^17^ announced that both aerobic MVPA and resistance training (RT) are highly recommended for adults with chronic conditions. RT upregulates the antioxidant capacity of skeletal muscles and balances the mitochondrial redox status^18^. A meta-analysis showed that RT improved respiratory symptoms, muscle strength, and lung function without any adverse events in patients with COPD^19^. In addition, aerobic MVPA + RT improved quality of life measured using the St George Respiratory Questionnaire total score as well as skeletal muscle strength in patients with COPD^19^. Another meta-analysis revealed that this combination is better for exercise capacity when compared to a non-exercising control group^20^. However, the effect of adding RT on respiratory symptoms and sleep quality in patients with COPD who already engage in regular aerobic MVPA remains unclear^7,21^.

Therefore, we hypothesized that compared with aerobic MVPA alone, aerobic MVPA + RT is associated with more adequate sleep duration and less respiratory symptoms, especially cough and sputum in male with airflow limitation.

## Results

The estimated number of men performing aerobic MVPA in the Korea National Health and Nutrition Examination Survey (KNHANES) during 2014–2019 was 4,614,521 (Table 1). The participants with aerobic MVPA alone were more likely to be current smokers compared with those with aerobic MVPA + RT, while those with aerobic MVPA + RT were more likely to live in urban area and have higher education and household income levels, compared with those performing aerobic MVPA alone.

**Table 1:** Characteristics of men with airflow limitation.

	MVPA alone (n = 480)	MVPA + RT (n = 314)	*P*
Population size	2,731,208	1,883,313	
Age	62.7 (0.6)	61.8 (0.7)	0.337
BMI (kg/m^2^)	23.9 (0.1)	24.3 (0.2)	0.087
Smoking			0.003
Never	14.2 (1.9)	16.9 (2.5)	
Former	45.9 (2.6)	57.5 (3.5)	
Current	39.9 (2.6)	25.6 (3.1)	
High-risk drinking^a^			0.119
No	57.6 (3.1)	65.4 (3.7)	
Yes	42.4 (3.1)	34.6 (3.7)	
Residence			0.009
Urban	80.7 (2.1)	88.9 (2.2)	
Rural	19.3 (2.1)	11.1 (2.2)	
Education			< 0.001
Middle school or lower	38.8 (2.7)	23.7 (2.7)	
High school	33.6 (2.6)	36.7 (3.3)	
College or higher	27.6 (2.6)	39.6 (3.4)	
Household income			0.026
Lowest	26.9 (2.3)	19.4 (2.5)	
Lower middle	25.2 (2.3)	19.7 (2.7)	
Higher middle	24.1 (2.3)	28.6 (3.4)	
Highest	23.7 (2.5)	32.4 (3.3)	
FEV_1_%pred	77.2 (0.8)	77.9 (0.9)	0.526
FEV_1_ < 80% pred	51.9 (2.8)	51.6 (3.3)	0.942
FEV_1_/FVC (%)	63.8 (0.3)	63.7 (0.4)	0.729
COPD diagnosis by physician	1.7 (0.7)	3.6 (1.0)	0.075

Values are presented as weighted means with standard errors for continuous variables and as weighted percentages with standard errors for categorical variables.

MVPA, moderate-to-vigorous physical activity; RT, resistance training; FEV_1_%pred = forced expiratory volume in one second of the predicted value; FVC, forced vital capacity; BMI, body mass index.

^a^High-risk drinking (seven [alcohol 60 g] or more drinks for men on one occasion).

**Figure 1:**
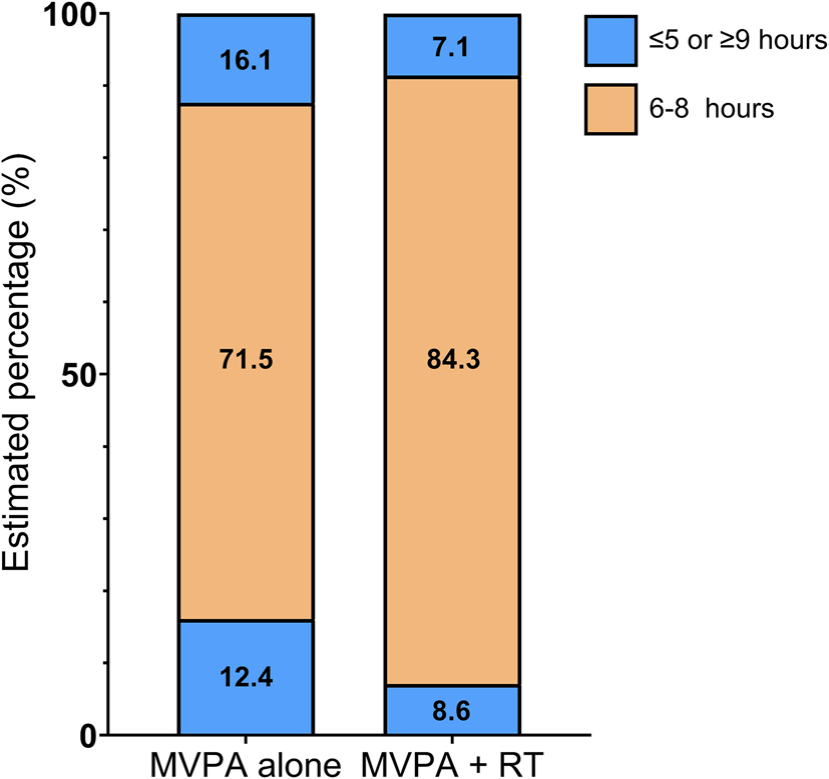
Estimated percentage (%) of sleep duration (hours) according to aerobic MVPA with RT and aerobic MVPA alone in male individuals with airflow limitation. MVPA, moderate-to-vigorous physical activity; RT, resistance training.

Unweighted numbers with percentages and weighted percentages with standard error (SE) of sleep problems were significantly lower in men with airflow limitation who performed aerobic MVPA + RT than in those who performed only aerobic MVPA (Table 2 and Fig. 1). The significance level was intensified in individuals with airflow limitation with a forced expiratory volume in 1 s (FEV_1_) < 80% of the predicted value (% pred). In complex sample logistic regression analysis, men with airflow limitation who underwent aerobic MVPA + RT had a significantly lower odds ratio (OR) for sleep problems than those who underwent only aerobic MVPA (OR 0.44, 95% confidence interval [CI]: 0.25–0.77, *P* = 0.004). The fully adjusted model revealed a 56% decrease in odds of the individuals with airflow limitation performing aerobic MVPA + RT compared with those performing only aerobic MVPA. However, this association was only significant in individuals with FEV_1_ < 80% pred, where the estimated OR with a 95% CI of sleep problems was 0.30 (0.15–0.63, *P* = 0.002).

**Table 2:** Numbers with percentages and odds ratio (OR) for sleep problems (≤ 5 or ≥ 9 h) in men with airflow limitation who performed both aerobic MVPA and RT compared to only aerobic MVPA.

	MVPA alone	MVPA + RT	*P*
Total			
Sleep problems, n (%)	139 (29.0%)	53 (16.9%)	< 0.001
Weighted % (SE)	28.5% (2.4)	15.7% (2.5)	0.001
OR (95% CI)			
Model 1	*Reference*	0.47 (0.29–0.73)	0.001
Model 2	*Reference*	0.43 (0.25–0.74)	0.002
Model 3	*Reference*	0.44 (0.25–0.78)	0.005
Model 4	*Reference*	0.44 (0.25–0.77)	0.004
FEV_1_ ≥ 80% pred			
Sleep problems, n (%)	65 (27.5%)	23 (15.4%)	0.006
Weighted % (SE)	26.9% (3.3)	15.8% (3.9)	0.04
OR (95% CI)			
Model 1	*Reference*	0.51 (0.27–0.97)	0.041
Model 2	*Reference*	0.58 (0.28–1.21)	0.145
Model 3	*Reference*	0.63 (0.28–1.43)	0.269
FEV_1_ < 80% pred			
Sleep problems, n (%)	74 (30.3%)	30 (18.2%)	0.006
Weighted % (SE)	30.0% (3.5)	15.6% (3.3)	0.006
OR (95% CI)			
Model 1	*Reference*	0.43 (0.24–0.79)	0.006
Model 2	*Reference*	0.31 (0.15–0.64)	0.002
Model 3	*Reference*	0.30 (0.15–0.63)	0.002

Model 1 was adjusted for age. Model 2 was additionally adjusted for body mass index, smoking status, and high-risk drinking status. Model 3 was additionally adjusted for residence, education level, and household income level. Model 4 was additionally adjusted for FEV_1_%pred.

MVPA, moderate-to-vigorous physical activity; RT, resistance training; FEV_1_%pred, forced expiratory volume in one second of the predicted value.; SE, standard error; CI, 95% confidence interval.

The unweighted number, weighted percentage, and odds of chronic cough for more than 3 months were significantly lower in men with airflow limitation who underwent aerobic MVPA + RT (Table 3). In all individuals with airflow limitation, fully adjusted model showed the OR of chronic cough was 0.35 (95% CI 0.13–0.94, *P* = 0.038). However, when subgrouping by FEV_1_% pred, this observation was only significant in the individuals with FEV_1_ < 80% pred (OR 0.23, 95% CI 0.06–0.84, *P* = 0.026). This pattern was similar to that observed in chronic sputum collected for more than 3 months (Table 4). After adjustment for all covariables, the OR of chronic sputum was 0.51 (95% CI 0.30–0.87, *P* = 0.012). However, when subgrouping by FEV_1_% pred, this observation was only significant in the individuals with FEV_1 <_ 80% pred (OR 0.49, 95% CI 0.25–0.99, *P* = 0.045).

**Table 3:** Unweighted numbers, weighted percentages, and odds ratios (OR) for chronic cough for more than three months within the past year in men with airflow limitation who performed both aerobic MVPA and RT compared to only aerobic MVPA.

	MVPA alone	MVPA + RT	*P*
Total			
Chronic cough, n (%)	40 (8.3%)	11 (3.5%)	0.007
Weighted % (SE)	7.6% (1.4)	2.6% (1.0)	0.008
OR (95% CI)			
Model 1	*Reference*	0.34 (0.14–0.80)	0.013
Model 2	*Reference*	0.37 (0.15–0.95)	0.039
Model 3	*Reference*	0.35 (0.13–0.93)	0.035
Model 4	*Reference*	0.35 (0.13–0.94)	0.038
FEV_1_ ≥ 80% pred			
Chronic cough, n (%)	8 (3.4%)	3 (2.0%)	0.43
Weighted % (SE)	3.3% (1.2)	2.1% (1.4)	0.558
OR (95% CI)			
Model 1	*Reference*	0.58 (0.11–3.20)	0.535
Model 2	*Reference*	0.68 (0.15–3.07)	0.616
Model 3	*Reference*	0.62 (0.18–2.22)	0.463
FEV_1_ < 80% pred			
Chronic cough, n (%)	32 (13.1%)	8 (4.8%)	0.006
Weighted % (SE)	11.6% (2.3)	3.1% (1.3)	0.003
OR (95% CI)			
Model 1	*Reference*	0.25 (0.09–0.67)	0.006
Model 2	*Reference*	0.27 (0.09–0.84)	0.023
Model 3	*Reference*	0.23 (0.06–0.84)	0.026

Model 1 was adjusted for age. Model 2 was additionally adjusted for body mass index, smoking status, and high-risk drinking status. Model 3 was additionally adjusted for residence, education level, and household income level. Model 4 was additionally adjusted for FEV_1_%pred.

MVPA, moderate-to-vigorous physical activity; RT, resistance training; FEV_1_%pred, forced expiratory volume within one second of the predicted value; SE, standard error; CI, 95% confidence interval.

**Table 4:** Unweighted numbers, weighted percentages, and odds ratios (OR) for chronic sputum for more than three months within past year in men with airflow limitation who performed both aerobic MVPA and RT compared to only aerobic MVPA.

	MVPA alone	MVPA + RT	*P*
Total			
Chronic sputum, n (%)	75 (15.6%)	27 (8.6%)	0.004
Weighted % (SE)	14.1% (1.8)	6.7% (1.3)	0.001
OR (95% CI)			
Model 1	*Reference*	0.45 (0.27–0.74)	0.001
Model 2	*Reference*	0.49 (0.30–0.82)	0.006
Model 3	*Reference*	0.51 (0.30–0.86)	0.011
Model 4	*Reference*	0.51 (0.30–0.87)	0.012
FEV_1_ ≥ 80% pred			
Chronic sputum, n (%)	29 (12.3%)	11 (7.4%)	0.124
Weighted % (SE)	11.8% (2.4)	5.5% (1.8)	0.034
OR (95% CI)			
Model 1	*Reference*	0.44 (0.20–0.99)	0.047
Model 2	*Reference*	0.53 (0.24–1.17)	0.116
Model 3	*Reference*	0.54 (0.23–1.29)	0.166
FEV_1_ < 80% pred			
Chronic sputum, n (%)	46 (18.9%)	16 (9.7%)	0.011
Weighted % (SE)	16.2% (2.4)	7.8% (1.9)	0.011
OR (95% CI)			
Model 1	*Reference*	0.44 (0.23–0.85)	0.014
Model 2	*Reference*	0.47 (0.24–0.94)	0.033
Model 3	*Reference*	0.49 (0.25–0.99)	0.045

Model 1 was adjusted for age. Model 2 was additionally adjusted for body mass index, smoking status, and high-risk drinking status. Model 3 was additionally adjusted for residence, education level, and household income level. Model 4 was additionally adjusted for FEV_1_%pred.

MVPA, moderate-to-vigorous physical activity; RT, resistance training; FEV_1_%pred, forced expiratory volume within one second of the predicted value; SE, standard error; CI, 95% confidence interval.

## Discussion

Using a large nationwide representative sample, we demonstrated that in men with airflow limitation, a combination of aerobic MVPA and RT were associated with decreased odds of sleep problems (either sleep deprivation of ≤ 5 h or oversleeping of ≥ 9 h), compared to aerobic MVPA alone. Interestingly, these findings were more pronounced in the individuals with a FEV_1_ < 80% pred. This observation is consistent with the finding that chronic cough and sputum production were significantly lower in men with airflow limitation who engaged in aerobic MVPA + RT than in those who performed only aerobic MVPA. Our study supports the most recent guidelines emphasizing the importance of not only aerobic MVPA but also RT in the daily life of individuals with chronic disease conditions, such as COPD, although further longitudinal studies are necessary.

In our study, 24.2% of participants suffered inadequate sleep duration. Poor sleep quality is common among COPD patients with respiratory symptoms^3–5^, however, various factors, such as physical disturbances, psychiatric problems, and environmental issues could contribute to inadequate sleep duration^22^. Sleep duration is one of the main aspects in assessing sleep disturbance and several studies have shown that regular exercise improves sleep quality^23,24^. In the general population, an analysis using NHANES data reported a quadratic relationship between aerobic MVPA and sleep duration^25^. Another population-based epidemiologic study showed that a combination of aerobic MVPA and RT is more beneficial for sleep quality, including appropriate sleep duration, than aerobic MVPA only^26^. Our study corroborates this finding by focusing on individuals with airflow limitation, revealing that aerobic MVPA + RT were more beneficial for adequate sleep duration than aerobic MVPA only. Given the lack of evidence to support COPD-specific recommendations on the role of aerobic MVPA and RT in appropriate sleep duration and the scarcity of evidence regarding this issue^7^, our study suggests the potential role of combined aerobic MVPA and RT on sleep quality.

One of the notable findings of this study is that aerobic MVPA + RT were associated with decreased odds of chronic cough and sputum in individuals with airflow limitation, compared to aerobic MVPA only. Patients with a high symptom burden are less likely to engage in regular daily aerobic MVPA, not only in patients with mild, but also severe airflow obstruction^15^. A recent community-based exercise intervention study revealed the feasibility of the role of PA in symptom relief in patients with COPD^27^. The study showed a 12-month efficacy and effectiveness of urban training which recommended ≥ 30 min of daily moderate PA for ≥ 5 days per week to increase PA levels and to improve respiratory symptoms and quality of life in patients with COPD^27^. Similarly, another randomized controlled trial including 102 patients with COPD reported that an intervention group received a standard PA program for 12 months, which improved their COPD Assessment Test and St. George’s Respiratory Questionnaire scores and increased their PA levels^28^. Our study extended this finding by showing that the addition of RT to aerobic MVPA was associated with decreased chronic cough and sputum production, supporting the recommendation of RT in addition to aerobic MVPA from the World Health Organization in 2020^16^ and the PA guidelines for Americans in 2018^17^. Furthermore, given that sleep disturbance is associated with respiratory symptoms including cough and sputum in patients with COPD^5,29^, our findings may provide further explanation for the improvement to adequate sleep duration with aerobic MVPA + RT.

Although a causal relationship could not be determined due to the cross-sectional study design, increased muscle strength and decreased prevalence of sarcopenia in individuals with airflow limitation performing aerobic MVPA + RT compared with those only performing aerobic MVPA partially account for the study’s findings (Supplementary Table 1). A meta-analysis of the general population discovered that sleep duration (under 6 h or more than 8 h) versus reference category (6–8 h) was significantly related to increased risk of sarcopenia^30^. Additionally, the presence of sarcopenia was related to poor quality of life in patients with COPD^12^. Meanwhile, the additional benefit of RT alongside aerobic MVPA might be minimal in individuals with COPD with relatively reserved lung function because they are less likely to have definite sleep disturbance or respiratory symptoms and are more likely to have increased muscle power and decreased proportion of sarcopenia. Thus, the impact of RT and aerobic MVPA may be more pronounced in patients with advanced airflow limitations.

Although our study suggests the importance of adding RT to aerobic MVPA in agreement with the existing PA guidelines from the World Health Organization and the PA Guidelines Advisory Committee of the United States^17^, it also has some limitations. First, this was a cross-sectional study, meaning that a causal relationship could not be determined. In other words, there is a possible explanation that adequate duration of sleep and less respiratory symptoms could lead to an active exercise. Future longitudinal studies are needed to clarify the additional role of RT in sleep, cough, and sputum production in patients with COPD. Second, as only pre-bronchodilator spirometry results are available in the KNHANES, further studies based on post-bronchodilator spirometry are necessary to validate our findings in COPD patients. Finally, our study was conducted only in males with airflow limitation. Thus, the results of the current study may not be generalizable to females with airflow limitation.

In conclusion, using large, nationally representative data, we demonstrated that a combination of RT and aerobic MVPA is associated with decreased inadequate sleep and respiratory symptoms such as chronic cough and chronic sputum in males with airflow limitation, compared to those who are engaged in only aerobic MVPA. This association was only significant for those who had an FEV_1_ < 80% pred, which implies the potential subgroup for whom it could be more beneficial. Further studies are necessary to elucidate the longitudinal impact of RT and aerobic MVPA in alleviating symptoms and improving sleep quality in patients of both sexes with COPD.

## Methods

### Datasets and study participants

This study used data from the KNHANES, a nationally representative survey of non-institutionalized Korean citizens conducted annually by the Korean Center for Disease Control and Prevention. The KNHANES collects data on socioeconomic status, health-related behaviors, anthropometric indices, and biochemical and clinical profiles of noncommunicable diseases. Each survey year includes a new sample of randomly enrolled participants. The detailed KNHANES procedures have been described previously^32^.

We collected data from KNHANES from 2014 to 2019, where of 61,754 total individuals, 47,309 (76.6%) responded to the survey. Since pulmonary function testing (PFT) was only performed in adults aged ≥ 40 years, participants aged < 40 years were excluded (n = 31085). Individuals without airflow limitation were also excluded (n = 13,595). Among the remaining 2629, 1763 men and 722 women were identified. Because our study purpose was to investigate the association of a combination of RT and aerobic MVPA with respiratory symptoms and sleep duration compared to aerobic MVPA alone, 1056 individuals (794 men and 262 women) who were engaged in aerobic MVPA were selected. However, of the 261 women, only 54 underwent additional RT. Therefore, our analysis was restricted to men with airflow limitation (n = 794).

### Spirometry

PFT was performed using dry rolling seal spirometers (Model 2130; SensorMedics, Yorba Linda, CA, USA) from 2014 to 2015 and Vyntus Spiro (CareFusion, San Diego, CA, USA) from 2016 to 2019. Calibration and quality control followed the standardization criteria of the American Thoracic Society and European Respiratory Society^33^. FEV_1_ (Liters), forced vital capacity (FVC, Liters), and the ratio of FEV_1_/FVC (%) were obtained from the pre-bronchodilator test. However, post-bronchodilator testing was not performed in the KNHANES.

Airflow limitation was defined as when pre-bronchodilator FEV_1_/FVC ratio is < 0.7. The GOLD recommends use of post-bronchodilator spirometry for COPD diagnosis^2^. Nonetheless, this operational definition of airflow limitation has been utilized in several major epidemiological studies representative for COPD, despite the possibility of overdiagnosis and no consideration of respiratory symptoms^34,35^. Severity of airflow limitation was classified as mild when the FEV_1_ was ≥ 80% pred and moderate-to-severe when the FEV_1_ was < 80% pred^2^.

### Aerobic MVPA and RT

The main exposure in this study was RT in addition to aerobic MVPA. Therefore, individuals who reported engaging in regular aerobic MVPA were further categorized based on whether they underwent RT. Attainment of aerobic MVPA was collected using self-reported questionnaires to assess regular aerobic exercise. A definition of aerobic MVPA was made when the following conditions were met: (1) vigorous-intensity PA for > 20 min per day on ≥ 3 days per week, (2) moderate-intensity PA for > 30 min per day on ≥ 5 days per week, or (3) an equivalent combination of moderate- and vigorous-intensity PA^17^. RT was assessed according to the number of days that exercises such as push-ups, sit-ups, use of dumbbell or weights, and chin-ups were performed per week and marked “yes” if the participant performed RT more than 2 days per week^17^.

### Sleep problems

Sleep duration was recorded using a self-reported questionnaire, assessed on weekdays and weekends separately, and the average sleep duration calculated. Adequate sleep was defined as having 6–8 h of sleep, and sleep problems were defined as when the individual showed sleep deprivation (sleep ≤ 5 h) or oversleep (sleep ≥ 9 h)^36–39^.

### Cough and sputum

Data on self-reported respiratory symptoms, specifically chronic cough and sputum production, were acquired using the following question: “Have you experienced sputum production or coughing persistently for a duration of more than three months within the past year?”. The answers options were either yes or no.

### Other variables

Hand grip strength (HGS) was measured using a digital hand dynamometer (Digital grip strength dynamometer, T.K.K 5401, Takei Scientific Instruments Co., Ltd., Tokyo, Japan). HGS was measured in the standing position with the forearm away from the body at the level of the thigh. A resting interval of at least 30 s was allowed between the measurements. HGS was defined as the mean value of the measured grip strength of the dominant hand. Sarcopenia was defined as a HGS < 28 kg in men^40^.

Other variables included BMI (kg/m^2^), smoking status (never, former, and current), high-risk drinking (seven [alcohol 60 g] or more drinks for men on one occasion), residence (rural or urban), education (middle school or lower, high school, and college or higher), and household income (lowest, lower middle, higher middle, and highest). The smoking status was categorized based on the National Health Interview Survey of the United States. Current smokers were defined as individuals who smoked more than 100 cigarettes in their lifetime and who currently smoked. Former smokers were defined as individuals who had smoked more than 100 cigarettes in their lifetime but had stopped smoking for more than 1 year.

### Statistical analysis

The KNHANES is designed to represent non-institutionalized South Korean citizens. To ensure representativeness, a stratified multistage probability sampling method was employed in the KNHANES design. Therefore, all statistical analyses conducted in this study utilized a complex sample analysis method, considering the sampling weights, stratification, and clustering of the KNHANES data.

Continuous variables were presented as weighted means and standard error (SE) and compared using complex sample linear regression analysis. Categorical variables were presented as weighted percentages with SE and compared using the chi-squared test.

Complex sample logistic regression analysis was used to estimate the weighted OR and 95% CI for sleep problems, chronic cough, and chronic sputum production. For multivariable analysis, Model 1 was adjusted for age; Model 2 was additionally adjusted for BMI, smoking status, and high-risk drinking status; Model 3 was additionally adjusted for residence, education, and household income level; and Model 4 was additionally adjusted for FEV_1_%pred.

Subgroup analyses were additionally conducted, stratified by the degree of airflow limitation (FEV_1_ ≥ 80 or < 80% pred).

All statistical analyses were performed using SPSS version 24 software Windows, Armonk, NY, USA). For all analyses, a *P* value < 0.05 was considered statistically significant.

### Ethical approval

The Institutional Review Board of Samsung Medical Center (no. 2023–09-028) approved the study and waived the requirement for informed consent because of the retrospective nature of this study and the KNHANES data were de-identified. The study was conducted in accordance with the principles of the Declaration of Helsinki. All procedures were performed in accordance with relevant guidelines and regulations.

### Supplementary Information

Supplementary Table 1.

## Data Availability

The datasets used and analyzed in the current study are available from the corresponding author upon reasonable request.
