# Supplementary material for: Association of resistance training and moderate-to-vigorous physical activity with clinical outcomes in men with airflow limitation: a nationwide population-based study
Source: Sci Rep. 2024 Mar 18;14:6436. doi: 10.1038/s41598-024-57232-6 (PMC10948750; doi:10.1038/s41598-024-57232-6)
Supplement: Supplementary file 1 — Supplementary Table 1. [file 41598_2024_57232_MOESM1_ESM.docx]

**Supplement Table 1.** Hand grip strength (HGS) and proportion of sarcopenia of male with airflow limitation

|  | **MVPA (n=480)** | **MVPA + RT (n=314)** | *P* |
| --- | --- | --- | --- |
| **HGS** |  |  |  |
| **Total** | 36.0 (0.4) | 38.2 (0.5) | 0.001 |
| FEV_1_ ≥ 80% pred | 37.1 (0.6) | 38.8 (0.7) | 0.07 |
| FEV_1_ < 80% pred | 34.9 (0.6) | 37.7 (0.7) | 0.005 |
| **Sarcopenia** |  |  |  |
| **Total** | 12.2% (1.7) | 6.2% (1.6) | 0.012 |
| FEV_1_ ≥ 80% pred | 7.9% (1.9) | 3.1% (1.3) | 0.036 |
| FEV_1_ < 80% pred | 16.0% (2.7) | 8.7% (2.6) | 0.068 |

HGS (kg) was estimated as mean values with SE for the dominant hand.

Sarcopenia was defined as HGS <28kg for males.

MVPA, moderate-to-vigorous physical activity; RT, resistance training; FEV_1_%pred, forced expiratory volume within one second of the predicted value; SE, standard error.
